# Total Hip Arthroplasty with a Cementless Acetabular Component and Impaction Bone Grafting for Dysplastic Osteoarthritis Complicated by Multiple Myeloma

**DOI:** 10.1155/2022/3939356

**Published:** 2022-02-12

**Authors:** Ryo Ogawa, Maki Hirao, Taro Umezu, Shigeru Yanagimoto

**Affiliations:** ^1^Department of Orthopaedic Surgery, Tokyo Saiseikai Central Hospital, 17-4-1 Mita, Minato, Tokyo, Japan; ^2^Department of Hematology, Tokyo Saiseikai Central Hospital, 17-4-1 Mita, Minato, Tokyo, Japan

## Abstract

Total hip arthroplasty (THA) for pathologic and impending fracture due to periacetabular multiple myeloma (MM) lesions has been reported. We report a case of radiographic progression of dysplastic osteoarthritis, complicated by periacetabular MM lesions, treated by THA. A 69-year-old female with a 13-year history of MM presented with right hip pain. Plain radiographs and CT showed that dysplastic osteoarthritis had progressed, while the periacetabular MM lesions remained unchanged. Pathologic fracture was not observed on MRI. THA with a cementless acetabular component and impaction bone grafting was done. Bone graft incorporation was confirmed on CT at 1 year after surgery. There were no signs of bone absorption or implant loosening at last follow-up 3 years after surgery. Due to the advances in the treatment of MM and antiresorptive drugs, cementless acetabular component and impaction bone grafting may be an option for dysplastic osteoarthritis complicated by acetabular bone loss due to MM.

## 1. Introduction

Multiple myeloma is a malignancy of monoclonal plasma cells, and it is the second most prevalent blood malignancy [1]. Bony involvement is common, and bony lesions due to MM tend to be more diffuse and more extensive than those from metastatic carcinoma [2]. The incidence of pelvic and periacetabular bony involvement in MM is reported to be around 6% [3]. Thanks to the advances in chemotherapy and stem cell transplantation, the median survival of MM has increased from 3 years to 6 years in the past two decades [4]. Thus, in the near future, more MM patients may require surgical treatment on hips complicated by periacetabular MM lesions.

In case series focusing on periacetabular MM lesions, the indications for surgery are either pathologic fracture or impending fracture [2, 5]. Here, we report a rare case of a 69-year-old female with right groin pain due to progression of dysplastic osteoarthritis of the hip. It was complicated by severe bone loss from periacetabular MM lesions and treated by THA using a cementless acetabular component and impaction bone grafting.

## 2. Case Report

69-year-old Asian female presented with a three-year history of right groin pain of gradual onset. At the age of 56, she was diagnosed with Bence-Jones *κ* type multiple myeloma (MM) and reached complete remission after undergoing autologous transplant. However, she relapsed one year ago, and salvage line chemotherapy and denosumab were started, and she reached very good partial response after 7 cycles.

**Figure 1:**
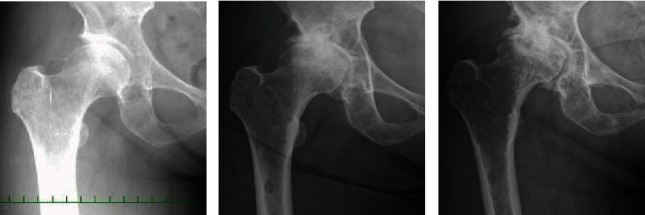
Plain radiographs, (a) 13 years prior to surgery, (b) 2 years prior to surgery, and (c) 1 month prior to surgery, reveal progression of arthritic changes including loss of joint space and femoral head cyst. Acetabular dysplasia with a center-edge angle of 13 degrees and sharp angle of 47 degrees, osteoarthritis with loss of joint space and bone cysts in the femoral head, and multiple myeloma with punched-out lesions are depicted.

**Figure 2:**
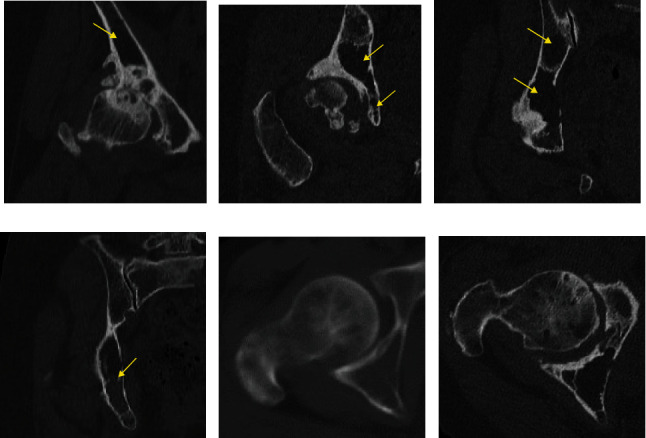
Coronal computed tomography (CT) images prior to surgery (a–d) reveal 20 × 35 mm cortical defect of the quadrilateral plate and massive bone absorption lesions of the cancellous bone in the supraacetabular region, medial wall, and ischium. The roof and anterior/posterior walls of the acetabulum are intact. The acetabular bone loss was classified as a Harrington class II defect. Axial CT images (e) 13 years prior to surgery and (f) 1 month prior to surgery reveal that bone absorption lesions existed 13 years ago.

**Figure 3:**
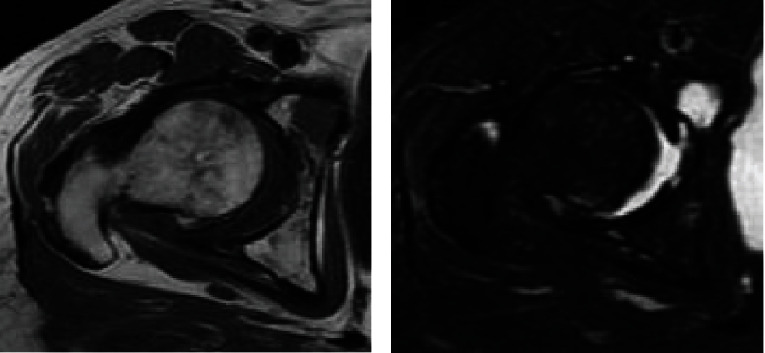
Nonenhanced (a) T1-weighted image and (b) T2-weighted image magnetic resonance image reveals multiple intraosseous lesions in the periacetabular region. There are no periacetabular pathologic fractures.

Her physical examination showed pain with hip joint motion in all directions and weight bearing and restricted range of motion. Harris hip score was 56. Plain radiographs showed acetabular dysplasia with a center-edge angle of 13 degrees and a sharp angle of 47 degrees; osteoarthritis with loss of joint space and bone cysts in the femoral head; and multiple myeloma with punched-out lesions in the femur, pelvic bone, and sacrum. Previous images revealed that osteoarthritic changes such as loss of joint space and femoral head bone cyst progressed over the past 13 years (Figure 1). CT revealed that the roof and anterior/posterior walls of the acetabulum were intact; however, there was a 20 mm by 35 mm cortical defect of the quadrilateral plate and massive bone absorption lesions of the cancellous bone in the supraacetabular region, medial wall, and ischium. The acetabular bone absorption lesions remained largely unchanged from 13 years ago (Figure 2). The acetabular bone loss was classified as class II by the Harrington classification and type I by the Paprosky classification. MRI revealed multiple intraosseous lesions in the femur, innominate bone, and sacrum; however, there were no pathologic fractures (Figure 3). The patient was diagnosed with progressive dysplastic osteoarthritis complicated by MM, rather than bone pain, impending fracture, or pathologic fracture.

**Figure 4:**
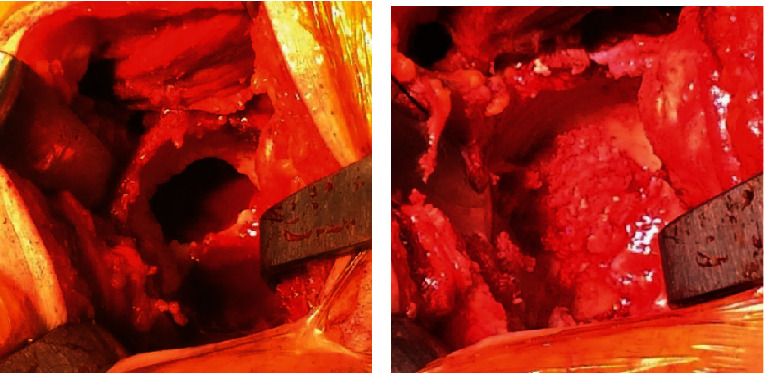
Intraoperative findings (a) after curettage of the acetabular bone absorption lesion through a window created in the acetabular dome and (b) after impaction bone grafting of the resulting void.

Survival prognosis was several years; therefore, surgical therapy was indicated. Total hip arthroplasty was performed through a posterolateral approach. The acetabular bone absorption lesion was thoroughly curettaged through a 20 mm by 20 mm window created in the acetabular dome. The resulting medial and superior bone defect was filled with a mixture of autogenous bone collected from the resected femoral head using a rounger and hydroxyapatite and then impacted with reverse reaming with a reamer 1 mm smaller than the final implant (Figure 4). A trabecular titanium cementless cup (SQRUM TT Cup, Kyocera, Kyoto, Japan) was implanted, and although initial press fit fixation of the cup was obtained on peripheral sclerotic bone, screws were inserted for supplemental initial stability. Taking into consideration the MM lesions in both the femoral metaphysis and diaphysis, a cemented femoral stem was implanted to minimize the risk of intraoperative fracture.

Immediate full-weight bearing was commenced postoperatively. Pain relief and full ambulation was achieved, and she was discharged 16 days after surgery without any perioperative complications. Chemotherapy and denosumab were continued postoperatively. Pathological examination of the surgically excised acetabular bone showed normocellular marrow with mild growth of *κ* type plasma myeloma cells, but there were no areas of diffuse proliferation, consistent with residual myeloma cells (data not shown).

**Figure 5:**
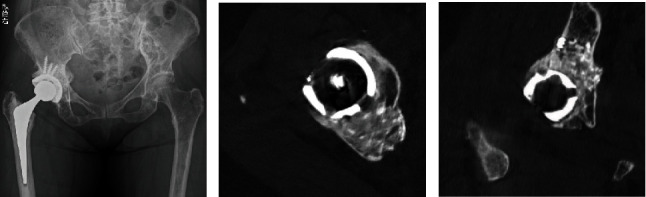
(a) Plain radiograph and CT images (b) axial and (c) coronal at 1 year after operation reveal incorporation of the acetabular bone grafts.

**Figure 6:**
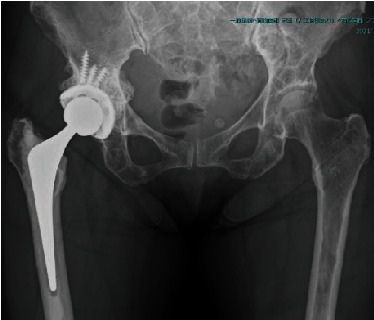
Plain radiograph at 3 years after surgery shows no signs of bone absorption or implant loosening.

At 1 year after surgery, Harris hip score was 93, and incorporation of the acetabular bone graft was confirmed on CT (Figure 5). At the last follow-up appointment 3 years after surgery, Harris hip score was 98, and there were no signs of bone absorption or implant loosening on plain radiograph (Figure 6).

## 3. Discussion

Multiple myeloma is the second most prevalent blood malignancy, and pelvis and periacetabular lesions in MM are reported to be around 6% [1, 3]. Pathologic fracture or impending fracture is the indication for surgery in case series focusing on periacetabular MM lesions [2, 5]. This is a case of progressive dysplastic hip osteoarthritis complicated by periacetabular bone loss due to MM. In regions where prevalence of hip dysplasia is high, more patients may need treatment of dysplastic osteoarthritis complicated by periacetabular MM lesions.

Harrington's classification, reported in 1981, is widely used to guide treatment for metastatic bone disease of the acetabulum, including MM lesions [3, 6, 7]. Class I lesions are contained cavitary defects with an intact roof, medial wall, and lateral cortices [6, 7]. In class II lesions, the medial wall and quadrilateral plate are deficient, but the roof and lateral cortices of the ilium, ischium, and pubis adjacent to the acetabulum are intact [6, 7]. With class III lesions, there are deficits in the medial wall, roof, and rim [6, 7].

Antiprotrusion ring with cementation of the medial wall has been the proposed management of Harrington's class II lesions in MM patients, due to the risk of central migration of the femoral head [3]. This treatment consists of methyl-methacrylate contained by medial wire mesh to fill the defect and an antiprotrusion ring to transfer weight bearing forces away from the medial wall [3, 8]. The use of cementless porous acetabular cups has been discouraged in metastatic lesions, due to the risk of local disease progression and the inhibitory effect on bone ingrowth by the disease and radiation therapy, which may result in postoperative implant instability and implant failure in the future [8].

However, several recent case series report no cases of prosthesis failure in series of metastatic hip lesions treated with cementless THA [9, 10]. Acetabular impaction bone grafting combined with cementless acetabular components is a well-established method, and is indicated for defects that can be contained, rendered contained, or when rim fixation can be achieved [11]. Bone autografts of lesion-free femoral heads have been used in pelvic metastases [2]. Bone lesions of MM are systemic, warranting the use of bone autografts of lesion-free sites.

In this case, initial fixation of an antiprotrusion ring was uncertain, due to osteolysis of the supraacetabular region. The cortical defect of the quadrilateral plate was positioned proximal to the level of the femoral head, making reinforcement with a metal wire mesh difficult. Also, cup supporters such as the KT plate and Burch-Schneider cage may not achieve stable fixation to host bone, since fixation with flanges, hooks, and screws into osteolytic ischium, teardrop, and ilium could be less stable.

Therefore, acetabular bone grafts combined with cementless acetabular components were judged to be more stable and less invasive than the combination of methyl-methacrylate and antiprotrusion ring. The acetabular rim and dome were sclerotic from osteoarthritis, enabling initial rim fixation of the acetabular component. Impaction bone grafting provided support for the acetabular dome and component, and transmits forces to intact proximal bone. Incorporation of the acetabular bone graft was achieved, providing long-term stability and increased bone strength.

A case of progressive dysplastic hip osteoarthritis complicated by periacetabular bone loss due to MM was treated with cementless acetabular component and impaction bone grafting. Although larger studies are needed to determine the long-term results of this treatment strategy, it may be a feasible option thanks to advances in the treatment of MM and advances in antiresorptive drugs.

## Data Availability

The study is a case report and there is no underlying data.
